# Vitamin D intake and determinants of vitamin D status during pregnancy in The Norwegian Mother, Father and Child Cohort Study

**DOI:** 10.3389/fnut.2023.1111004

**Published:** 2023-06-23

**Authors:** Anna Amberntsson, Linnea Bärebring, Anna Winkvist, Lauren Lissner, Helle Margrete Meltzer, Anne Lise Brantsæter, Eleni Papadopoulou, Hanna Augustin

**Affiliations:** ^1^Department of Internal Medicine and Clinical Nutrition, Institute of Medicine, Sahlgrenska Academy, University of Gothenburg, Gothenburg, Sweden; ^2^School of Public Health and Community Medicine, Institute of Medicine, Sahlgrenska Academy, University of Gothenburg, Gothenburg, Sweden; ^3^Department of Food Safety, Division of Climate and Environmental Health, Norwegian Institute of Public Health, Oslo, Norway; ^4^Global Health Cluster, Division of Health Services, Norwegian Institute of Public Health, Oslo, Norway

**Keywords:** determinants, vitamin D intake, 25-hydroxyvitamin D, pregnancy, The Norwegian Mother, Father and Child Cohort Study (MoBa)

## Abstract

**Background:**

Norwegian data on vitamin D status among pregnant women indicate a moderate to high prevalence of insufficient vitamin D status (25-hydroxyvitamin D (25OHD) concentrations ≤50  nmol/L). There is a lack of population-based research on vitamin D intake and determinants of 25OHD in pregnant women from northern latitudes. The aims of this study were (1) to evaluate total vitamin D intake from both diet and supplements, (2) to investigate determinants of vitamin D status, and (3) to investigate the predicted response in vitamin D status by total vitamin D intake, in pregnant Norwegian women.

**Methods:**

In total, 2,960 pregnant women from The Norwegian Environmental Biobank, a sub-study within The Norwegian Mother, Father and Child Cohort Study (MoBa), were included. Total vitamin D intake was estimated from a food frequency questionnaire in gestational week 22. Concentrations of plasma 25OHD was analyzed by automated chemiluminescent microparticle immunoassay method in gestational week 18. Candidate determinant variables of 25OHD were chosen using stepwise backward selection and investigated using multivariable linear regression. Predicted 25OHD by total vitamin D intake, overall and stratified by season and pre-pregnancy BMI, was explored using restricted cubic splines in an adjusted linear regression.

**Results:**

Overall, about 61% of the women had a total vitamin D intake below the recommended intake. The main contributors to total vitamin D intake were vitamin D supplements, fish, and fortified margarine. Higher 25OHD concentrations were associated with (in descending order of the beta estimates) summer season, use of solarium, higher vitamin D intake from supplements, origin from high income country, lower pre-pregnancy BMI, higher age, higher vitamin D intake from foods, no smoking during pregnancy, higher education and energy intake. During October–May, a vitamin D intake according to the recommended intake was predicted to reach sufficient 25OHD concentrations >50  nmoL/L.

**Conclusion:**

The findings from this study highlight the importance of the vitamin D intake, as one of few modifiable determinants, to reach sufficient 25OHD concentrations during months when dermal synthesis of vitamin D is absent.

## 1. Introduction

The importance of vitamin D throughout life is widely acknowledged, although it is likely that some of its biological functions have not yet been discovered. Vitamin D is obtained from dermal synthesis induced by solar exposure and intake from foods and supplements (1, 2). After endogenous synthesis or absorption, vitamin D is hydroxylated in a two-step process, formation of: (1) the biomarker 25-hydroxyvitamin D (25OHD) and (2) the biologically active metabolite 1,25-dihydroxyvitamin D (1,25OH2D) (3). The main role of 1,25OH2D is to ensure adequate concentrations of calcium and phosphate in plasma (3, 4). However, in early pregnancy, the vitamin D metabolism change and maternal concentrations of 1,25OH2D rise, provided that maternal concentration of 25OHD is sufficient (5, 6). The reason for the alterations in vitamin D metabolism is not entirely clear (6, 7). Besides skeletal health (8), vitamin D has also been studied for its potential associations with a range of detrimental maternal and neonatal outcomes, including preeclampsia, gestational diabetes, and low birth weight (9, 10). For these associations, the evidence of causality is insufficient.

Despite the many potential health effects, recommended concentrations of the biomarker 25OHD are based on the role of 1,25OH2D for skeletal health (11–13). In the Nordic Nutrition Recommendations 2012 (NNR 2012), a 25OHD concentration >50 nmol/L define sufficiency, 30–50 nmol/L define insufficiency, and < 30 nmol/L define deficiency, in both pregnant and non-pregnant individuals (13). To achieve concentrations of 25OHD >50 nmol/L, the NNR 2012 set the average requirement (AR) and the recommended intake (RI) of vitamin D for adults, including pregnant women, to 7.5 μg/day and 10 μg/day, respectively. An intake of 20 μg/day is recommended for those with little or no sun exposure, or who are older than 75 years of age. In Norway, the adult population is recommended to supplement with 10 μg/day of vitamin D if sun exposure and the intake of vitamin D rich foods are low (14, 15). Pregnant women in Norway are given the advice to supplement with vitamin D if fatty fish consumption is less than 2–3 times/week (16). Fish and eggs are some of the foods that naturally contain vitamin D, while margarine, butter, and some fat-reduced milk are fortified with vitamin D in Norway (15).

No study has previously investigated a broad spectrum of determinants of vitamin D status, also including the vitamin D intake from foods and supplements in a pregnant Norwegian population. As dermal synthesis of vitamin D can only occur from late spring (April–May) to early autumn (August–September) in the Nordic countries (17), sufficient vitamin D intake is important to maintain adequate vitamin D status during the rest of the year. Due to increased calcium requirements during pregnancy, and possible negative effects of insufficient vitamin D status on maternal and neonatal outcomes, the importance of vitamin D sufficiency during pregnancy is highlighted. Thus, there is a need to increase the understanding of how vitamin D intake and other determinants contribute to 25OHD in pregnant women living in Nordic countries. The aims of this study were (1) to evaluate total vitamin D intake from both diet and supplements, (2) to investigate determinants of vitamin D status, and (3) to investigate the predicted response in vitamin D status by total vitamin D intake, in pregnant Norwegian women.

## 2. Materials and methods

### 2.1. Study population

The Norwegian Mother, Father and Child Cohort Study (MoBa) is a prospective population-based pregnancy cohort study conducted by the Norwegian Institute of Public Health (18). Participants were recruited from all over Norway from 1999 to 2008, and 41% of the invited women consented to participate. The cohort now includes approximately 114,500 children, 95,200 mothers and 75,200 fathers. MoBa data are linked to information from the Medical Birth Registry of Norway, a national health registry containing information about all births in Norway (19). The Norwegian Environmental Biobank is a sub-study within MoBa (20), established with the aim of biomonitoring nutrients and environmental contaminants in MoBa participants. Eligibility of participants in the Norwegian Environmental Biobank required available genetic data, available data from the three questionnaires during pregnancy and the first three postnatal questionnaires, the fathers’ questionnaire (21), and available maternal plasma, urine, and whole blood samples. In total, 2,999 women from MoBa were included in the Norwegian Environmental Biobank. Of these 2,988 had the biomarker 25OHD analyzed in pregnancy and were thus eligible for inclusion in the current study. Further, 28 participants (0.9%) with implausible reported daily energy intake below 1,076 kcal (4.5 MJ) or above 4,780 kcal (20 MJ) were excluded from the analyses. These cut-points are used in MoBa to exclude food frequency questionnaires (FFQs) of poor quality and are based on results from the validation study (22). Thus, the final analytic sample consisted of 2,960 pregnant women.

MoBa is conducted according to the guidelines laid down in the declaration of Helsinki and written informed consent was obtained from all participants. The establishment of MoBa and initial data collection was based on a license from the Norwegian Data Protection Agency and approval from The Regional Committees for Medical and Health Research Ethics. MoBa is currently regulated by the Norwegian Health Registry Act. The current study was approved by The Regional Committees for Medical and Health Research Ethics (REC 2019/770–12172). The current study is based on version 12 of the quality-assured data files released for research in January 2019 (23).

### 2.2. Assessment of dietary intake and vitamin D supplement use

The women’s habitual food consumption and supplement use since becoming pregnant was assessed by a semi-quantitative FFQ including 255 food items answered in gestational week 22 (22, 24, 25). Intake frequency of each item in the FFQ ranged from never to more than eight times a day. Portion size was only specified for liquids, bread, and fruit, while standard Norwegian portion sizes for women were used for all other food items (26). Food intake (g/day) and energy intake (kcal/day) was estimated based on the given intake frequency and portion size. At the time of data collection, the following foods were fortified with vitamin D: margarine and butter (8.0 μg/100 g), fat-reduced milk (0.4 μg/100 g), and lactose free milk (0.4 μg/100 g). In the current study, all foods containing vitamin D were aggregated into eight food groups: fish, margarine, butter, milk, yoghurt, eggs, cheese, and mixed dishes/products containing milk, margarine, butter, and/or egg. The women’s intake of these foods (g/day), as well as their contribution to the daily vitamin D intake (μg/day) were calculated. The women’s intake of vitamin D from supplements was also calculated, based on the frequency and amount of supplement intake reported in the FFQ. The FFQ listed 13 commonly used cod liver oil/fish oil, vitamin, and mineral supplements followed by six open-ended spaces where respondents were instructed to record the name and manufacturer of supplement(s) used but not listed. For each supplement there were nine options for frequency, ranging from never to daily, and for dose there were three options for liquid supplements and four options for tablets/capsules. For nutrient calculation of the dietary supplements, a database containing the nutrient value of more than 1,000 different food supplements was constructed and continuously updated (24, 27).

The estimated vitamin D intake from foods and supplements has previously been validated in a sub-group of 119 women using a weighed food diary and biomarkers. The results showed fair agreement between reported vitamin D intake and 25OHD with Spearman’s rank correlation coefficient (0.32–0.45, *p* < 0.01) and <5% classified into opposing quintiles of vitamin D intake and 25OHD (24, 25).

### 2.3. Assessment of vitamin D status in plasma

The blood samples were collected in gestational week 18 (mean 18.5, SD 1.3) in connection with the routine ultrasound examination, offered to all pregnant women in Norway (20). Samples were analyzed at the National Institute for Health and Welfare in Helsinki, Finland. Plasma concentrations of 25OHD were analyzed in 2015 using the high through-put automated chemiluminescent microparticle immunoassay method (Architect ci8200 system, Abbott Laboratories, Abbott Park, IL, United States). Both 25OHD2 and 25OHD3 are measured and the sum is reported. The laboratory regularly partake in the Vitamin D External Quality Assessment Scheme and met the performance target set by the advisory panel (28). Coefficient of variation of control samples (Biorad Liquid Assayed Multiqual lot 45,680 high and low level) were 3.7–5.5%.

### 2.4. Other variables

Maternal age at time of delivery, parity, and country of origin was obtained from the Medical Birth Registry of Norway. Information about education, pre-pregnant weight, height, smoking habits, and use of solarium was obtained from questionnaires, answered around gestational weeks 15 and 30. In gestational weeks 15, participants were also asked about use of vitamin D containing supplements (yes or no) by weekly intervals prior to conception, and in the first trimester.

### 2.5. Statistical analysis

Dietary intake and supplement use during the first half of pregnancy were described by categories of 25OHD, based on the reference values (<30 nmol/L, 30–49.9 nmol/L, 50–75 nmol/L, >75 nmol/L) in NNR 2012 (13) and National Academy of Medicine (Institute of Medicine) (12). Statistical difference in study population characteristics between the categories of 25OHD were assessed by Kruskal Wallis test.

The candidate determinant variables of vitamin D status were selected based on biological plausibility and included: season at blood sampling (two seasons; June–September, October–May), vitamin D intake from foods (<2.5, 2.5–4.9, 5.0–7.49, ≥7.5 μg/day), supplemental vitamin D intake (none, 0.1–4.9, 5.0–9.9, 10.0–14.9, ≥15.0 μg/day), energy intake (kcal/day), use of solarium during pregnancy (no, 1–5, ≥6 times), country of origin (Norway, other high income country, low/middle income country), education (<13, 13–16, >16 years), pre-pregnancy BMI (<18.5, 18.5–24.9, 25.0–29.9, ≥30.0 kg/m^2^), age (<25, 25–34, >34 years), smoking during pregnancy (yes, no), and parity (nulliparous, multiparous). The variables were checked for multicollinearity using Spearman correlation matrix, and all correlation coefficients were below 0.4. Variables were selected using stepwise backward selection (29) and *p* < 0.2 as cut-off for inclusion. Bootstrap subsampling was used to investigate and quantify the stability of selected variables (30). One hundred resamples were drawn and variable selection was repeated in each of the resamples. Variables included in >50% of the resamples were selected in the final model (30). No variable was excluded after the stability check. Results are presented as a full sample and stratified by season, as season is the main source of vitamin D and that there is a large seasonal variation in 25OHD concentrations in the Nordic countries (17), and pre-pregnancy BMI. The estimates of the determinants of 25OHD concentration in pregnancy were investigated using multivariable linear regression analysis.

The association between the total vitamin D intake and continuous 25OHD, in all women and stratified by season and pre-pregnancy BMI, was explored using restricted cubic splines. Three splines were modeled for the total vitamin D intake, positioned at percentiles 10, 50, and 90 (31). Models were adjusted for country of origin, age, and smoking during pregnancy. The models in all women and stratified by season were additionally adjusted for pre-pregnancy BMI. The overall associations between total vitamin D intake and 25OHD were performed by testing the coefficients of all spline transformations equal to zero. Non-linearity was examined by testing the coefficients of the second spline transformation equal to zero. The associations between use of vitamin D containing supplements before and during pregnancy and maternal concentrations of 25OHD were analyzed by Kruskal Wallis test.

Stata version 16 was used for all statistical analyses (Stata Corporation, College Station, Texas). Significance was accepted at *p* < 0.05.

## 3. Results

### 3.1. Study population

Of the 2,960 participants, 72% had a high education (≥13 years) and 5.9% reported smoking during pregnancy (Table 1). Only 4.8% were born in a high-income country other than Norway, and 1.4% were born in a low or middle-income country. There were differences between vitamin D categories with regard to the characteristics of the study population in terms of, e.g., age, education, country of origin, and pre-pregnancy BMI. About 80% of the participants reported use of vitamin D supplement in pregnancy. Around 44% of all women had a total vitamin D intake below AR at 7.5 μg/day and 61% had an intake below RI at 10 μg/day (13). Median (25^th^–75^th^ percentiles) 25OHD concentration was 51 (38–64) nmol/L.

**Table 1:** Study population characteristics by category of vitamin D status (25OHD) during pregnancy.

		Vitamin D status (25OHD) nmol/L				
	All	<30	30–50	>50–75	>75	*p*-value^b^
	% (*N*)	% (*N*)	% (*N*)	% (*N*)	% (*N*)	
Women	100 (2960)	10.9 (323)	37.4 (1106)	40.9 (1211)	10.8 (320)	
Age (years)						0.026
<25	8.0 (238)	17.6 (42)	37.0 (88)	39.1 (93)	6.3 (15)	
25–34	75.6 (2237)	10.3 (230)	37.7 (844)	41.1 (920)	10.9 (243)	
>34	16.4 (485)	10.5 (51)	35.9 (174)	40.8 (198)	12.8 (62)	
Education (years)						0.003
<13	25.8 (764)	13.9 (106)	37.7 (288)	40.4 (309)	8.0 (61)	
13–16	46.9 (1388)	9.9 (137)	35.7 (496)	42.9 (595)	11.5 (160)	
>16	25.2 (745)	9.3 (69)	39.6 (295)	39.1 (291)	12.1 (90)	
Missing	2.1 (63)	17.5 (11)	42.9 (27)	25.4 (16)	14.3 (9)	
Country of origin^a^						<0.001
Norway	92.0 (2723)	10.4 (283)	37.0 (1007)	41.5 (1129)	11.2 (304)	
Other high-income country	4.8 (141)	14.9 (21)	41.8 (59)	34.8 (49)	8.5 (12)	
Low/middle-income country	1.4 (42)	33.3 (14)	38.1 (16)	26.1 (11)	2.4 (1)	
Missing	1.8 (54)	9.3 (5)	44.4 (24)	40.7 (22)	5.6 (3)	
Pre-pregnancy BMI (kg/m^2^)						<0.001
<18.5	2.7 (79)	12.7 (10)	21.5 (17)	51.9 (41)	13.9 (11)	
18.5–24.9	63.7 (1886)	8.7 (164)	36.1 (680)	42.4 (800)	12.8 (242)	
25–29.9	24.1 (712)	13.3 (95)	39.2 (279)	40.2 (286)	7.3 (52)	
≥30	7.7 (229)	19.7 (45)	48.5 (111)	27.9 (64)	3.9 (9)	
Missing	1.8 (54)	16.7 (9)	35.2 (19)	37.0 (20)	11.1 (6)	
Parity						0.020
Nulliparous	51.3 (1519)	9.6 (146)	36.4 (553)	43.3 (657)	10.7 (163)	
Multiparous	48.7 (1441)	12.3 (177)	38.4 (553)	38.4 (554)	10.9 (157)	
Season of blood sampling						<0.001
June–September	30.2 (893)	3.2 (29)	22.4 (200)	53.8 (480)	20.6 (184)	
October–May	69.8 (2086)	14.1 (294)	43.4 (906)	35.0 (731)	6.5 (136)	
Solarium in pregnancy (times)						<0.001
No	86.5 (2569)	11.8 (304)	38.7 (994)	38.9 (1000)	9.6 (247)	
1–5	11.5 (343)	4.7 (16)	29.2 (100)	49.3 (169)	16.3 (56)	
≥6	2.0 (59)	3.4 (2)	16.9 (10)	54.2 (32)	23.7 (14)	
Vitamin D supplement use (≥0.1 μg/day)						<0.001
Yes	80.4 (2379)	9.0 (214)	35.9 (854)	43.2 (1028)	11.9 (283)	
No	9.0 (266)	18.0 (48)	41.0 (109)	35.0 (93)	6.0 (16)	
Missing	10.6 (315)	19.4 (61)	45.4 (143)	28.6 (90)	6.6 (21)	

25OHD, 25-hydroxyvitamin D; BMI, Body Mass Index.

a Based on the Global Burden of Disease super regions classification system (32).

b Statistical difference between the categories of 25OHD were assessed by Kruskal Wallis test.

### 3.2. Vitamin D from foods and supplements

**Figure 1:**
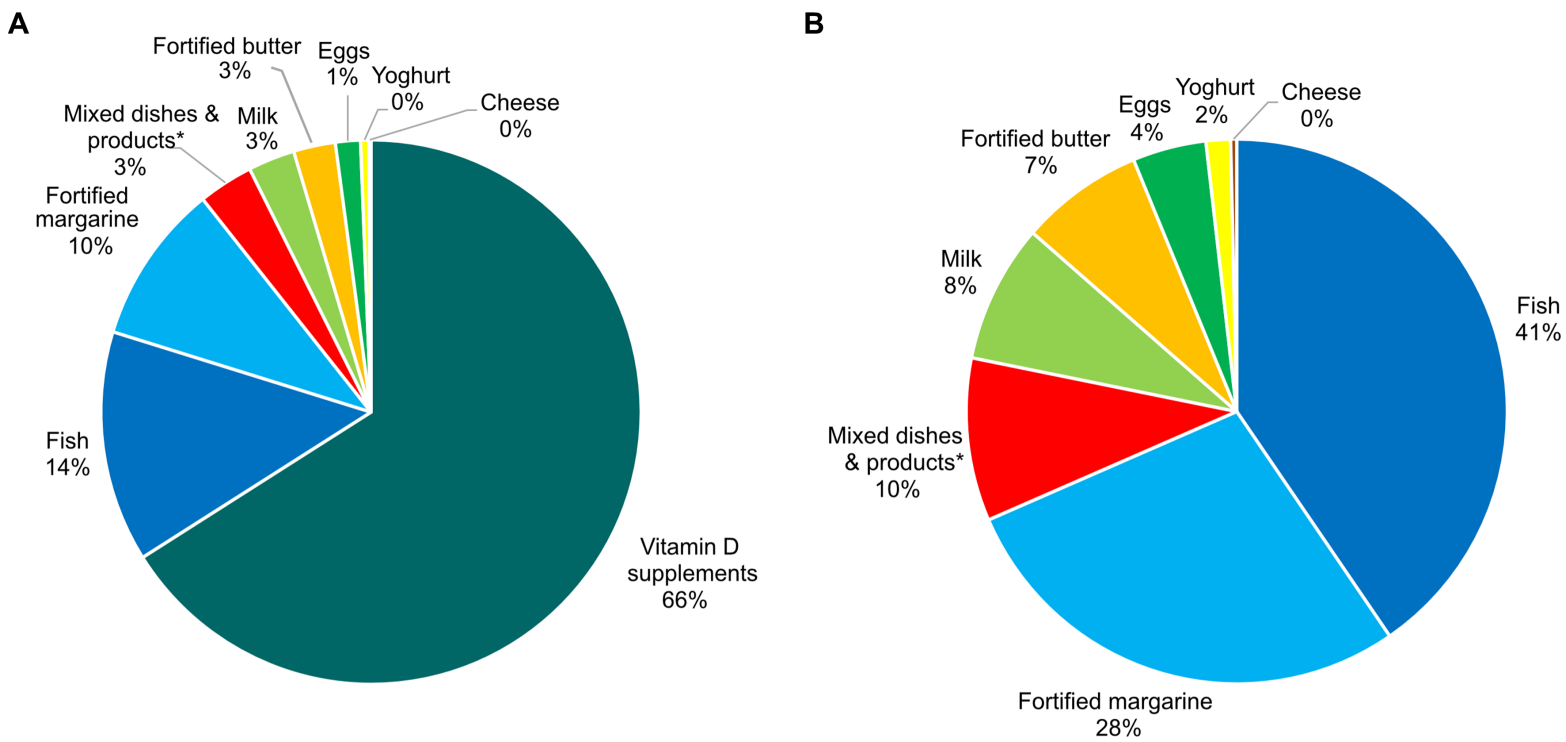
Sources contributing to the vitamin D intake in pregnancy in the Norwegian Environmental Biobank (*N* = 2,960), with **(A)** and without **(B)** contribution from vitamin D supplements. *Including milk, fortified margarine and butter, and/or egg.

Overall, the median vitamin D intake from foods was 3.1 μg/day, and the total vitamin D intake (from foods and supplements combined) was 8.3 μg/day (Table 2). Thus, the main contributor to vitamin D intake in pregnancy was vitamin D supplements (66%, Figure 1). The main food sources of vitamin D were fish and fortified margarine.

**Table 2:** Dietary intake and supplement use by categories of vitamin D status during pregnancy.

	Vitamin D status (25OHD) nmol/L					
	All	<30	30–50	>50–75	>75	*p*-value^a^
Women, *N*	2960	323	1106	1211	320	
Reported energy intake, kcal/day	2204 (1876, 2610)	2186 (1914, 2598)	2201 (1866, 2592)	2214 (1877, 2639)	2182 (1898, 2606)	0.854
Total vitamin D intake, μg/day^b^	8.3 (5.2, 13.1)	6.2 (3.7, 8.9)	7.8 (4.8, 11.6)	9.1 (5.5, 14.3)	10.5 (6.4, 17.1)	<0.001
Supplemental vitamin D intake, μg/day	5.0 (2.2, 9.8)	2.6 (0.9, 5.6)	4.3 (1.5, 7.9)	5.4 (2.6, 10.0)	7.2 (2.6, 13.6)	<0.001
Supplemental vitamin D intake, μg/day (users only)^c^	5.2 (2.6, 10.0)	4.0 (2.1, 6.3)	5.0 (2.6, 8.6)	6.3 (2.6, 11.4)	7.5 (3.2, 14.1)	<0.001
Vitamin D intake from food, μg/day	3.1 (2.1, 4.4)	2.9 (1.9, 4.0)	3.1 (2.1, 4.3)	3.2 (2.2, 4.5)	3.4 (2.2, 4.4)	<0.001
Vitamin D from fish, µg/day	1.1 (0.6, 1.8)	1.1 (0.6, 1.7)	1.1 (0.6, 1.7)	1.2 (0.6, 1.8)	1.1 (0.7, 1.8)	0.094
Vitamin D from margarine, µg/day	0.6 (0.1, 1,7)	0.2 (0.0, 1.4)	0.5 (0.1, 1.7)	0.8 (0.1, 1.7)	0.6 (0.0, 1.7)	0.038
Vitamin D from butter, µg/day	0.03 (0.00, 0.11)	0.02 (0.00, 0.12)	0.03 (0.00, 0.11)	0.02 (0.00, 0.11)	0.03 (0.00, 0.12)	0.888
Vitamin D from milk, µg/day	0.002 (0.00, 0.06)	0.002 (0.000, 0.026)	0.002 (0.000, 0.058)	0.002 (0.000, 0.055)	0.002 (0.002, 0.116)	0.133
Vitamin D from yoghurt, µg/day	0.025 (0.007, 0.075)	0.025 (0.012, 0.075)	0.025 (0.007, 0.053)	0.025 (0.007, 0.075)	0.025 (0.007, 0.075)	0.491
Vitamin D from eggs, µg/day	0.11 (0.05, 0.11)	0.11 (0.05, 0.11)	0.11 (0.05, 0.11)	0.11 (0.05, 0.01)	0.11 (0.05, 0.15)	0.370
Vitamin D from cheese, µg/day	0.008 (0.002, 0.016)	0.007 (0.002, 0.015)	0.007 (0.002, 0.015)	0.010 (0.002, 0.017)	0.011 (0.003, 0.018)	0.006
Vitamin D from mixed dishes and products, µg/day^d^	0.2 (0.2, 0.3)	0.2 (0.2, 0.4)	0.2 (0.2, 0.3)	0.2 (0.2, 0.3)	0.2 (0.16, 0.3)	0.390
Nutrient intake, g/day						
Protein	83 (72. 97)	82 (70. 97)	83 (71. 96)	84 (73. 98)	85 (73. 97)	0.178
Added sugar	51 (35, 74)	52 (37, 80)	51 (35, 73)	52 (36, 75)	48 (33, 69)	0.174
Fiber	29 (24, 36)	30 (24, 36)	29 (24, 36)	29 (24, 36)	29 (24, 36)	0.905
Food intake, g/day						
Fish	36 (23, 50)	36 (22, 50)	37 (23, 52)	36 (22, 50)	34 (23, 49)	0.328
Milk	288 (87, 428)	228 (82, 415)	313 (85, 428)	267 (87, 430)	400 (142, 477)	0.045
Yoghurt	32 (12, 89)	25 (12, 88)	32 (12, 85)	35 (12, 102)	37 (12, 103)	0.069
Margarine	13 (1, 24)	10 (1, 21)	13 (1, 24)	15 (2, 25)	14 (1, 25)	0.011
Butter	0.05 (0.0, 0.6)	0.05 (0.0, 0.6)	0.05 (0.0, 0.6)	0.05 (0.0, 0.5)	0.05 (0.0, 0.6)	0.805
Eggs	8 (4, 8)	8 (4, 8)	8 (4, 8)	8 (4, 8)	8 (4, 11)	0.370
Cheese	18 (9, 33)	18 (9, 36)	18 (9, 32)	18 (9, 32)	19 (9, 34)	0.509
Fruit and vegetables	389 (265, 546)	395 (264, 559)	384 (256, 538)	388 (269, 542)	402 (284, 561)	0.330

Values are provided as median (25th, 75th percentiles). 25OHD, 25-hydroxyvitamin D.

a Statistical difference between the categories of 25OHD were assessed by Kruskal Wallis test.

b Dietary and supplemental vitamin D intake.

c All; *N* = 2,379, <30 nmol/L; *N* = 214, 30–50 nmol/L; *N* = 854, >50–75 nmol/L; *N* = 1,028, >75 nmol/L; *N* = 283.

d Including milk, fortified margarine and butter, and/or egg.

### 3.3. Determinants of vitamin D status

There was a difference in vitamin D intake from both foods and supplements between categories of 25OHD concentration, but no differences were observed for intake of energy or other nutrients (Table 2). Among the major food sources of vitamin D, there was a difference. In the consumption of margarine, but not fish or butter, between categories of 25OHD.

In a multivariable linear regression, determinants of 25OHD concentrations in pregnancy were (in descending order of the beta estimates) summer season, use of solarium, higher vitamin D intake from supplements, origin from high income country, lower pre-pregnancy BMI, higher age, higher vitamin D intake from foods, no smoking during pregnancy, higher education and energy intake (Table 3). Together, these variables explained 21% of the variation in 25OHD. During June to September, total vitamin D intake explained 1% of the variation in 25OHD, whereas during October to May, it was 7% (Table 3). Stratified by pre-pregnancy BMI, the total vitamin D intake explained 5% of the variation in 25OHD in women with pre-pregnancy BMI <25 kg/m^2^, whereas in women with pre-pregnancy BMI ≥25 kg/m^2^, it was 3% (Supplementary Table S1).

**Table 3:** Determinants of vitamin D status (25OHD, nmol/L) in pregnancy.

	Vitamin D status (25OHD) nmol/L					
	All *N* = 2,476		June–September *N* = 738		October–May *N* = 1,738	
	Beta	*p*-value	Beta	*p*-value	Beta	*p*-value
Season of blood sampling						
June–September	Ref					
October–May	−14.13	<0.001				
Vitamin D intake from foods (μg/day)						
<2.5	Ref		Ref		Ref	
2.5–4.9	2.00	0.010	0.34	0.831	2.56	0.003
5.0–7.49	3.43	0.003	2.80	0.221	3.58	0.008
≥7.5	5.25	0.009	3.87	0.366	5.62	0.013
Supplemental vitamin D intake (μg/day)						
None	Ref		Ref		Ref	
0.1–4.9	3.86	0.010	3.36	0.148	4.12	0.003
5.0–9.9	6.02	<0.001	4.23	0.080	6.64	<0.001
10.0–14.9	10.23	<0.001	6.70	0.018	11.71	<0.001
≥15.0	13.34	<0.001	7.10	0.010	15.82	<0.001
Energy intake (kcal/day)	−0.002	0.004	−0.002	0.146	−0.002	0.023
Use of solarium in pregnancy (times)						
No	Ref		Ref		Ref	
1–5	6.03	<0.001	4.43	0.039	6.70	<0.001
≥ 6	13.37	<0.001	6.23	0.243	15.48	<0.001
Country of origin						
Norway	Ref		Ref		Ref	
Other high-income country	−2.18	0.179	−2.37	0.500	−2.05	0.255
Low/middle-income country	−10.53	0.001	−18.91	<0.001	−4.83	0.204
Education (years)						
<13	Ref		Ref		Ref	
13–16	1.89	0.029	3.02	0.085	1.51	0.129
>16	0.53	0.598	2.09	0.304	−0.17	0.883
Pre-pregnancy BMI (kg/m^2^)						
<24.9	Ref		Ref		Ref	
25–29.9	−4.83	<0.001	−5.34	0.001	−4.77	<0.001
≥30	−9.62	<0.001	−11.08	<0.001	−8.61	<0.001
Age (years)						
<25	Ref		Ref		Ref	
25–34	3.84	0.003	4.35	0.079	3.28	0.030
>34	5.75	<0.001	6.14	0.044	5.59	0.002
Smoking in pregnancy						
No	Ref		Ref		Ref	
Yes	−3.20	0.044	−4.63	0.095	−2.51	0.204
Parity						
Nulliparous	Ref		Ref		Ref	
Multiparous	0.26	0.720	2.74	0.057	−0.65	0.432

Results are shown for all women and stratified by season at blood sampling.

25OHD, 25-hydroxyvitamin D; BMI, Body Mass Index; Ref, reference category.

### 3.4. Total vitamin D intake and 25OHD

**Figure 2:**
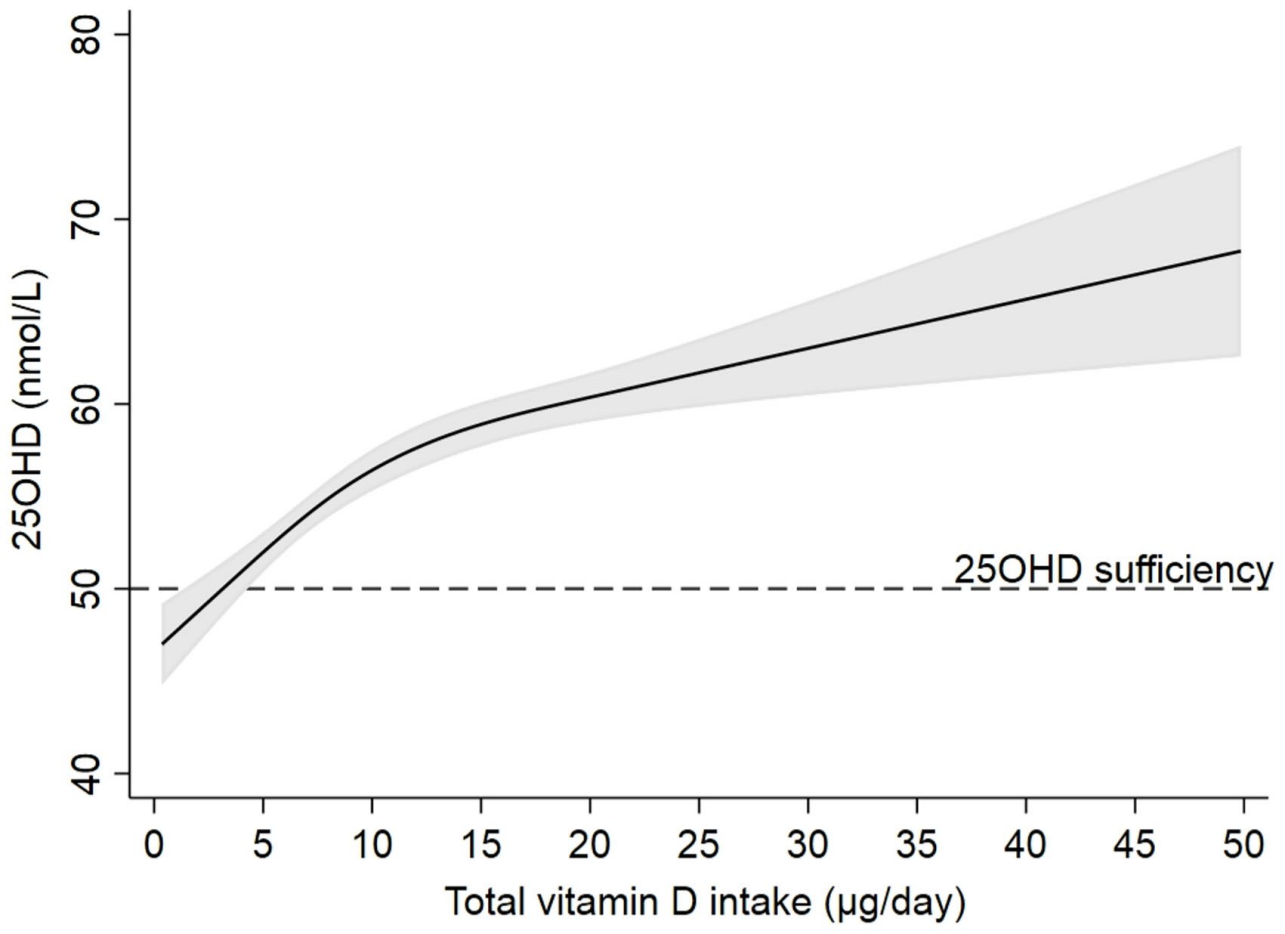
Predictions of 25-hydroxyvitamin D (solid black line) and 95% CI (grey area) by total vitamin D intake after estimating a linear regression model (*N* = 2,645) using restricted cubic splines, adjusted for country of origin, pre-pregnancy BMI, age, and smoking during pregnancy. Knots were placed at 3.5, 8.3, and 19.5 μg/day. Dotted black line corresponds to 25OHD sufficiency (50  nmol/L).

In the full sample of women, total vitamin D intake >5 μg/day was sufficient to reach predicted 25OHD >50 nmol/L (*p*-overall: <0.001, *p* non-linearity: <0.001, Figure 2). The effect of vitamin D intake on 25OHD seemed to level off at reported intakes >15 μg/day.

**Figure 3:**
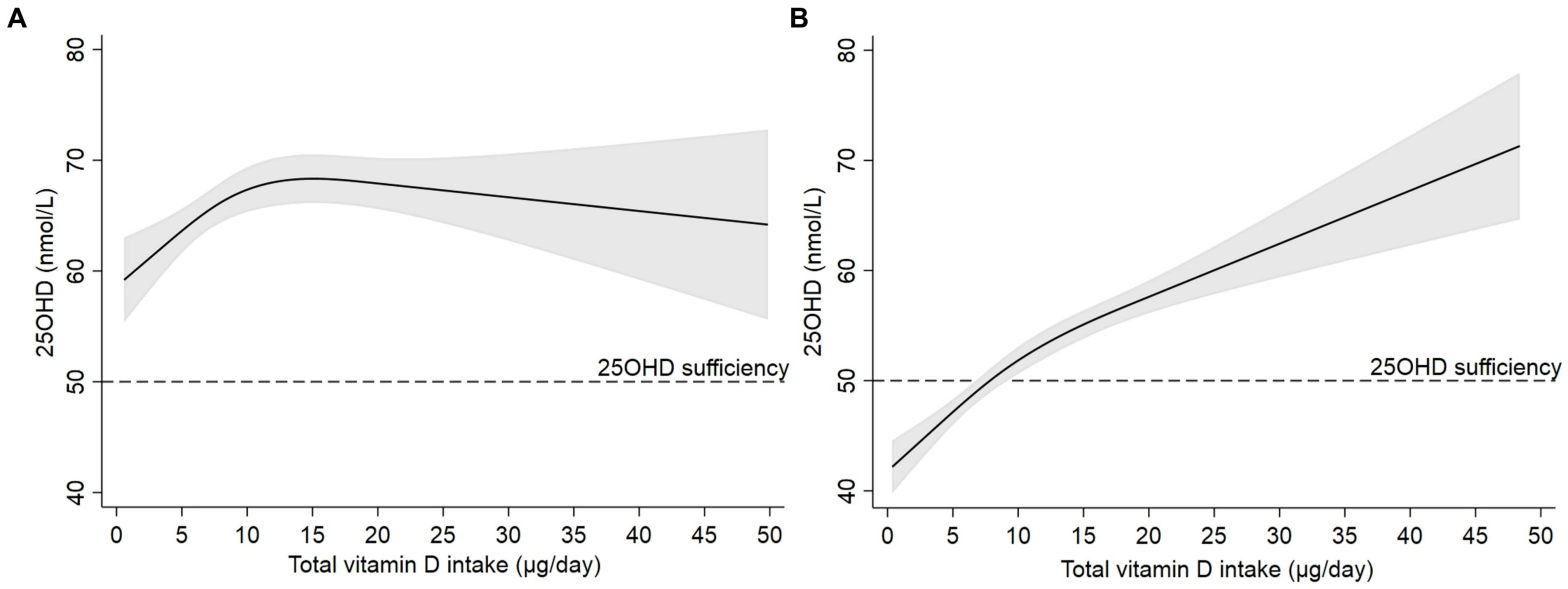
Predictions of 25-hydroxyvitamin D (solid black line) and 95% CI (grey area) by total vitamin D intake after estimating a linear regression model using restricted cubic splines in women blood sampling during **(A)** June to September (*N* = 796) and **(B)** October to May (*N* = 1,849). Models were adjusted for country of origin, pre-pregnancy BMI, age, and smoking during pregnancy. Knots were placed at 3.5, 8.3, and 19.5  μg/day. Dotted black line corresponds to 25OHD sufficiency (50  nmol/L).

When total vitamin D intake was stratified by season of blood sampling, women who were sampled during June to September had a predicted 25OHD >50 nmol/L even at very low intakes (*p*-overall: 0.001, *p* non-linearity: 0.003, Figure 3A). However, during October to May, a total vitamin D intake ≥10 μg/day was required to reach predicted 25OHD >50 nmol/L (*p*-overall: <0.001, *p* non-linearity: 0.023, Figure 3B).

When total vitamin D intake was stratified by pre-pregnancy BMI, women with BMI <25 kg/m^2^ had predicted 25OHD >50 nmol/L at vitamin D intakes ≥5 μg/day (*p*-overall: <0.001, *p* non-linearity: 0.004, Supplementary Figure S1A). Women with BMI ≥25 kg/m^2^ required a vitamin D intake ≥15 μg/day to reach a predicted sufficient 25OHD (*p*-overall: <0.001, *p* non-linearity: 0.043, Supplementary Figure S1B).

There was a difference in 25OHD concentrations depending on duration and timing of vitamin D supplement use during pregnancy (Supplementary Table S2). Women who reported any use of vitamin D supplement, either before and/or in early pregnancy, or in mid pregnancy, or both, had a higher 25OHD compared to women who reported no use of vitamin D supplement either before or during pregnancy. Women who reported use of vitamin D supplement both before and/or in early pregnancy and in mid pregnancy had a higher 25OHD compared to those who started to take a supplement in mid pregnancy.

## 4. Discussion

There is a lack of studies investigating the contribution of vitamin D intake and other determinants of 25OHD in pregnant Nordic women. In this study of 2,960 participants, about 61% had a total vitamin D intake below the RI and about 44% had an intake below the AR. The main contributors to the vitamin D intake were vitamin D supplements, fish, and fortified margarine. The most important determinants of vitamin D status were (in descending order of the beta estimates) season, solarium use, vitamin D intake from supplements, country of origin, pre-pregnancy BMI, age, vitamin D intake from foods, and smoking during pregnancy. During October to May, a vitamin D intake according to the RI was predicted to ensure sufficient 25OHD concentration.

The results from this study emphasize the important contribution of vitamin D supplements to reach RI of vitamin D and as a determinant of 25OHD. More than half of the women in our study had a total vitamin D intake below the RI, and supplements were the main contributor to the total vitamin D intake. Vitamin D intake from foods and supplements in our study was somewhat lower compared to other studies of pregnant women in Norway, Sweden, and Finland (33–36). In Sweden and Finland, more foods are fortified with vitamin D and with higher amounts than in Norway, which might partly explain the differences. A study on pregnant women in Norway found the vitamin D intake from foods and supplements was 4.9 and 5.6 μg/day, respectively (34). The intake from foods was higher compared to our study. However, the supplemental intake was similar to our study, along with a similar prevalence (59%) of a vitamin D intake below RI. Different dietary assessment methods might explain the differences in vitamin D intakes. Our results also show that women who reported any use of vitamin D supplement had a higher 25OHD compared to women who reported no use of vitamin D supplement, further highlighting the importance of vitamin D supplements during pregnancy.

The nationwide diet survey Norkost 3 from 2010–2011 found, in both women and men, that the major food sources contributing with vitamin D were fish (40%), followed by fortified margarine and butter (30%), eggs (17%), cakes (6%), and milk (4%), estimated by repeated 24-h recalls (37). The contribution from fish and margarine was similar to the results from our study, while the contribution of vitamin D from eggs was higher. However, Norkost 3 included both women and men and a wider age range, and the results are therefore not fully comparable.

We were able to explain 21% of the variation in 25OHD during pregnancy by both lifestyle factors and non-modifiable factors. The determinants with largest effect on the beta estimates were (in descending order) season at blood sampling, solarium use, vitamin D intake from supplements, country of origin, pre-pregnancy BMI, age, vitamin D intake from foods, smoking during pregnancy, education, and energy intake. A multi-ethnic study in Oslo, Norway, found 46% of the variation in 25OHD concentrations in gestational week 15 being explained by country of origin, season of blood sampling, and supplemental vitamin D intake ≥10 μg (38). In gestational week 28, the degree of explanation was lower (38) and similar to the results in our study. Other studies from Nordic countries have also found vitamin D intake from foods (33, 39) and supplements (33, 39) or total vitamin D intake (40), season (33, 39), ethnicity (40), age (39), BMI (39), smoking during pregnancy, solarium use, and outdoor physical activity (39) as determinants of 25OHD in pregnancy. Latitude and sunlight exposure are some factors known to affect the 25OHD concentration (41). These variables were not available though. However, solarium use might possibly also reflect other behaviors related to sun exposure, such as clothing habits and preference of sun or shade. This might have affected the effect estimates related to solarium use.

Pre-pregnancy BMI has previously been identified as a determinant of 25OHD in pregnancy, in some (39, 42), but not all (38) univariate models. Pre-pregnancy BMI modified the predicted response in 25OHD by total vitamin D intake. In addition, vitamin D intake from foods was not a significant determinant of 25OHD in women with pre-pregnancy BMI ≥25 kg/m^2^. These findings may indicate a lower response in 25OHD in women with BMI ≥25 kg/m^2^ by vitamin D intake or it might be due to reporting bias. Even though the causality is not fully understood, there is a negative association between 25OHD concentrations and body fat (43), possibly by sequestering of vitamin D in adipose tissue (44).

We also investigated the effect of the total vitamin D intake on 25OHD and found a plateau effect in intakes >15 μg/day. Possible explanations for this could be falsely overreporting of high vitamin D intakes, lack of data in this high intake category, or that the dose–response effect may be weaker at high 25OHD concentrations.

Using an immunoassay method and unstandardized values, almost half of the women in our study was classified as having either insufficient or deficient concentrations of 25OHD. Another study on pregnant women in Norway found that the prevalence of 25OHD <50 nmol/L was 47% and 25OHD <30 nmol/L was observed in 11% (45). A study on pregnant women in Denmark found that 10% had 25OHD <25 nmol/L and 42% had 25OHD <50 nmol/L (39). In pregnant women living in Sweden, 10% had 25OHD concentrations <30 nmol/L and 25% had 25OHD <50 nmol/L (40). Differences in vitamin D status have been found between ethnic groups (38, 46), at different latitudes (34), and by season (40, 47), which can explain differences in 25OHD concentrations between studies in Nordic populations, along with differences between laboratories and 25OHD assay methods (48).

We excluded 28 women (0.9%) due to implausibly low or high energy intake. Median (25^th^–75^th^ percentile) 25OHD concentration of the excluded women were 47 (32–54) nmol/L and median pre-pregnancy BMI were 23.3 (21.0–25.9) kg/m^2^. As so few were excluded, it has likely not impacted our results.

### 4.1. Strengths and limitations of the study

The main strength of our study is the availability of numerous potential determinants of vitamin D in a relatively large study population. In addition, questionnaires provided information both prospectively and retrospectively during pregnancy. The FFQ can be considered a suitable dietary assessment method in this study since only few foods contain significant amounts of vitamin D making food diaries less suitable and since the FFQ provides a fair estimation of the habitual diet over a specific period (49).

Some limitations should be considered in the interpretation of the results. Some studies have observed underestimation of 25OHD using Abbott Architect chemiluminescence immunoassays compared with liquid chromatography tandem mass spectrometry (50, 51), also in a pregnant population (52). Standardization of 25OHD was not possible as there was no plasma left for such analysis. If the assay method underestimated the 25OHD concentrations in our study, it would potentially lead to biased estimates of insufficiencies and deficiencies. Thus, the prevalence of vitamin D insufficiency and deficiency should be interpreted with caution. Further, some important variables were not available to us in the investigation of determinants of 25OHD, such as recent travels to southern latitudes and portion sizes. The participants included in the Norwegian Environmental Biobank were women with good compliance in MoBa. Thus, selection bias may be present and might negatively affect the representativeness and the external validity of the results. In addition, the lack of variation in country of origin in the study population limits the interpretation of the vitamin D intake and status in ethnic minorities. Other limitations related to the FFQ used in MoBa have previously been described (22, 25).

### 4.2. Implications

This study provides a thorough investigation of the vitamin D intake and other determinants of vitamin D status in pregnant women living in Norway. The contribution of the determinants is likely relevant to pregnant women also in other northern regions on corresponding latitudes. In addition, the response in 25OHD by total vitamin D intake might differ by season and pre-pregnancy BMI. Future studies should aim to investigate how vitamin D intake and status can be safely increased on a population level.

## 5. Conclusion

The results emphasize the importance of vitamin D from supplements, fish, and fortified margarine contributing to the total vitamin D intake. The most important determinants of vitamin D status were season, solarium use, vitamin D intake from supplements, country of origin, pre-pregnancy BMI, age, vitamin D intake from foods, and smoking during pregnancy. Our study indicates a seasonal variation in vitamin D status during pregnancy, and a dose–response in 25OHD by total vitamin D intake during October to May. These findings highlight the importance of the vitamin D intake, as one of few modifiable determinants, to reach sufficient 25OHD concentrations, during months when dermal synthesis of vitamin D is absent.

## Data availability statement

Data from MoBa used in this study are owned and managed by a third-party organization, the national health register holders in Norway (Norwegian Institute of Public Health). Researchers who want access to data sets from MoBa for replication should apply to www.helsedata.no/en. Access to data sets requires approval from The Regional Committee for Medical and Health Research Ethics in Norway and an agreement with MoBa.

## Ethics statement

The studies involving human participants were reviewed and approved by The Regional Committees for Medical and Health Research Ethics in Norway. The patients/participants provided their written informed consent to participate in this study.

## Author contributions

HA initiated the study. HA, LB, AA, EP, AB, LL, HM, and AW planned the study and were involved in interpretation of the results and writing of the final manuscript. HA and AB are responsible for data protection and access. AA conducted the statistical analyses and wrote the first version of the manuscript. HA, LB, and EP assisted with the statistical analyses. All authors contributed to the article and approved the submitted version.

## Funding

The current study was funded by the Swedish Research Council for Health, Working Life and Welfare (grant number 2018-00441) and the Sahlgrenska Academy (U2018/162). The Norwegian Mother, Father and Child Cohort Study is supported by the Norwegian Ministry of Health and Care Services and the Ministry of Education and Research. The Norwegian Institute of Public Health has contributed to funding of the Norwegian Environmental Biobank. The funders had no role in study design, data collection and analysis, decision to publish, or preparation of the manuscript.

## Conflict of interest

The authors declare that the research was conducted in the absence of any commercial or financial relationships that could be construed as a potential conflict of interest.

## Publisher’s note

All claims expressed in this article are solely those of the authors and do not necessarily represent those of their affiliated organizations, or those of the publisher, the editors and the reviewers. Any product that may be evaluated in this article, or claim that may be made by its manufacturer, is not guaranteed or endorsed by the publisher.
