# Supplementary material for: Vitamin D intake and determinants of vitamin D status during pregnancy in The Norwegian Mother, Father and Child Cohort Study
Source: Front Nutr. 2023 Jun 23;10:1111004. doi: 10.3389/fnut.2023.1111004 (PMC10327547; doi:10.3389/fnut.2023.1111004)
Supplement: Supplementary file 1 [file Data_Sheet_1.docx]

Supplementary Material

Vitamin D intake and determinants of vitamin D status during pregnancy in the Norwegian Mother, Father and Child Cohort Study

**Anna Amberntsson^1^*, Linnea Bärebring^1^, Anna Winkvist^1^, Lauren Lissner^2^, Helle Margrete Meltzer^3^, Anne Lise Brantsæter^3^, Eleni Papadopoulou^4^, Hanna Augustin^1^**

*** Correspondence:** Anna Amberntsson: anna.amberntsson@gu.se

**Supplementary Table** **1.** Determinants of vitamin D status (25OHD, nmol/L) in pregnancy stratified by pre-pregnancy BMI.

|  | Vitamin D status (25OHD) nmol/L | | | |
| --- | --- | --- | --- | --- |
|  | Pre-pregnancy BMI  <25 kg/m^2^  N=1,718 | | Pre-pregnancy BMI  ≥25 kg/m^2^ N=758 | |
|  | **Beta** | **P-value** | **Beta** | **P-value** |
| Season of blood sampling |  |  |  |  |
| June-September | Ref |  | Ref |  |
| October-May | -14.42 | <0.001 | -13.35 | <0.001 |
| Vitamin D intake from foods (µg/day) |  |  |  |  |
| <2.5 | Ref |  | Ref |  |
| 2.5-4.9 | 2.78 | 0.004 | 0.48 | 0.697 |
| 5.0-7.49 | 4.71 | 0.001 | 1.00 | 0.628 |
| ≥7.5 | 6.95 | 0.006 | 1.54 | 0.640 |
| Supplemental vitamin D intake (µg/day) |  |  |  |  |
| None | Ref |  | Ref |  |
| 0.1-4.9 | 3.21 | 0.034 | 5.36 | 0.007 |
| 5.0-9.9 | 5.69 | <0.001 | 6.34 | 0.002 |
| 10.0-14.9 | 9.72 | <0.001 | 11.13 | <0.001 |
| ≥15.0 | 13.61 | <0.001 | 12.11 | <0.001 |
| Energy intake (kcal/day) | -0.002 | 0.016 | -0.002 | 0.123 |
| Use of solarium in pregnancy (times) |  |  |  |  |
| No | Ref |  | Ref |  |
| 1-5 | 4.81 | <0.001 | 9.76 | <0.001 |
| ≥6 | 10.53 | <0.001 | 23.35 | <0.001 |
| Country of origin |  |  |  |  |
| Norway | Ref |  | Ref |  |
| Other high-income country | -1.58 | 0.412 | -4.45 | 0.149 |
| Low/middle-income country | -9.52 | 0.018 | -11.16 | 0.014 |
| Education (years) |  |  |  |  |
| <13 | Ref |  | Ref |  |
| 13-16 | 1.73 | 0.126 | 2.52 | 0.058 |
| >16 | 0.04 | 0.977 | 2.42 | 0.147 |
| Age (years) |  |  |  |  |
| <25 | Ref |  | Ref |  |
| 25-34 | 3.02 | 0.070 | 5.09 | 0.014 |
| >34 | 4.66 | 0.020 | 7.36 | 0.003 |
| Smoking in pregnancy |  |  |  |  |
| No | Ref |  | Ref |  |
| Yes | -5.18 | 0.012 | 0.79 | 0.750 |
| Parity |  |  |  |  |
| Nulliparous | Ref |  | Ref |  |
| Multiparous | 0.22 | 0.810 | 0.28 | 0.808 |

Abbreviations: 25OHD, 25-hydroxyvitamin D; BMI; Body Mass Index, Ref; reference category

**Supplementary Table 2.** Reported use of vitamin D supplements before and during pregnancy by vitamin D status assessed in mean gestational week 18.

| **Vitamin D supplement use in pregnancy** | | **Maternal 25OHD** | | **Number in  sub-group** |
| --- | --- | --- | --- | --- |
| Pre and early pregnancy ^a^ | Mid pregnancy ^b^ | Mean (SD) | Median (p25, p75) * | N (%) |
| No | No | 47.5 (19.4) | 45 (34, 58) | 995 (33.6) |
| Yes | No | 50.2 (18.2) | 49 (37, 62.5) | 344 (11.6) |
| No | Yes | 51.8 (19.1) | 50 (38, 65) | 354 (12.0) |
| Yes | Yes | 56.3 (18.1) | 55 (43, 67) | 1267 (42.8) |

Abbreviations: 25OHD, 25-hydroxyvitamin D; p, percentile; SD, Standard Deviation
^a^ 8 weeks before pregnancy to gestational week 12
^b^ Gestational weeks 13-20
* P<0.001. Statistical difference in 25OHD between the categories of vitamin D supplement use were assessed by Kruskal Wallis test

**
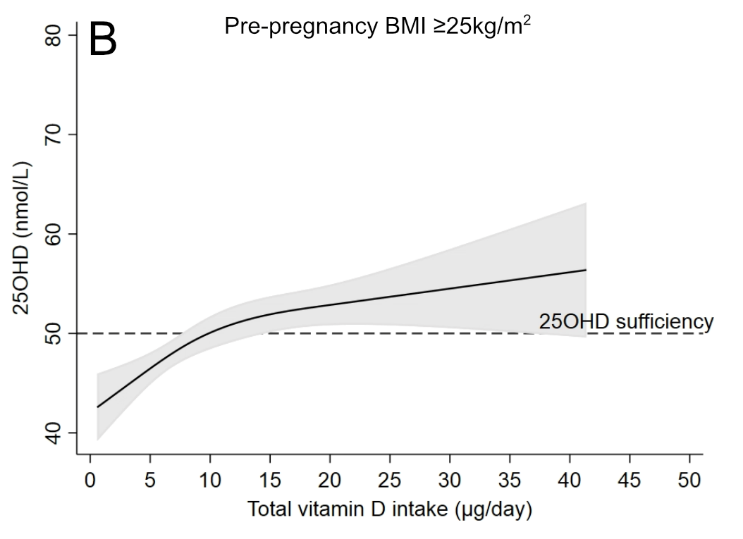

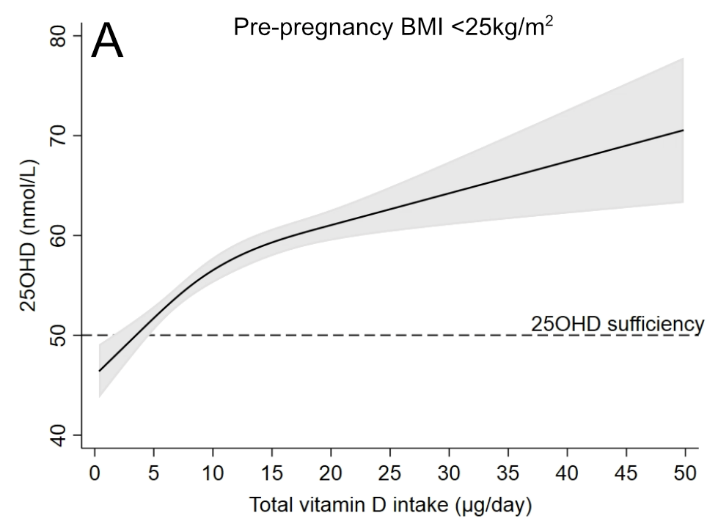
**

**Supplementary Figure 1**. Predicted 25OHD (solid black line) and 95% CI (grey area) by total vitamin D intake. Knots were placed at 3.5, 8.3, and 19.5 µg/day in a linear regression in (A) women with pre-pregnancy BMI <25kg/m^2^ (N=1,794), and (B) women with pre-pregnancy BMI ≥25 kg/m^2^ (N=803). Models were adjusted for country of origin, age, and smoking during pregnancy.
